# The genome sequence of the black-veined white butterfly, *Aporia crataegi* (Linnaeus, 1758)

**DOI:** 10.12688/wellcomeopenres.17709.1

**Published:** 2022-03-08

**Authors:** Sam Ebdon, Alexander Mackintosh, Konrad Lohse, Alex Hayward, Saad Arif, Rebecca Whitla, Darwin Tree of Life Barcoding collective, Wellcome Sanger Institute Tree of Life programme, Wellcome Sanger Institute Scientific Operations: DNA Pipelines collective, Tree of Life Core Informatics collective, Darwin Tree of Life Consortium

**Affiliations:** 1 Institute of Evolutionary Biology, University of Edinburgh, Edinburgh, UK; 2 Department of Biosciences, University of Exeter, Penryn, UK; 3 Department of Biological and Medical Sciences, Oxford Brookes University, Headington, Oxford, UK

**Keywords:** Aporia crataegi, black-veined white, genome sequence, chromosomal, Lepidoptera

## Abstract

We present a genome assembly from an individual male *Aporia crataegi* (the black-veined white; Arthropoda; Insecta; Lepidoptera; Pieridae). The genome sequence is 230 megabases in span. The complete assembly is scaffolded into 26 chromosomal pseudomolecules, with the Z sex chromosome assembled. Gene annotation of this assembly on Ensembl has identified 10,860 protein coding genes.

## Species taxonomy

Eukaryota; Metazoa; Ecdysozoa; Arthropoda; Hexapoda; Insecta; Pterygota; Neoptera; Endopterygota; Lepidoptera; Glossata; Ditrysia; Papilionoidea; Pieridae; Pierinae; Aporia; *Aporia crataegi* (Linnaeus, 1758) (NCBI:txid111923).

## Background

The black-veined white ( *Aporia crataegi*) is a large butterfly with distinctive venation on its wings. This species is oligophagous with a larval host plant preference for *Prunus* and *Crataegus* spp *.* and is often considered a pest species in orchards ( Jugovic *et al.,* 2017; Manley, 2008). It is found in a wide variety of habitats including dry grassland, woodland edges, and shrubland ( Tolman & Lewington, 2008). *Aporia crataegi* is found across the Palaearctic, with populations present in northwest Africa, as well as across Europe and Asia. The butterfly disappeared from the British Isles around 1925, and the last British specimens were collected from Herne Bay in Kent during the 1920s ( Todisco *et al.,* 2020). It is not understood why the species disappeared from the British Isles; however, climate variability along with other concurrent detrimental conditions, such as parasites, disease, or predation have been suggested as potential reasons ( Pratt, 1983). Several reintroductions have been attempted, but all have been unsuccessful ( Asher *et al.,* 2001), including one purportedly by Winston Churchill after the end of World War II. Given the butterfly's wide Palaearctic distribution, it remains listed as a species of least concern, but more recently it has been reported as extinct in the Czech Republic, the Netherlands ( Van Swaay *et al.*, 2010), and likely South Korea ( Kim *et al.,* 2015). Additionally, abundance and/or range is declining in Austria, Luxembourg, Romania, Ukraine, Albania, France, Latvia, Norway and Serbia ( Van Swaay *et al.,* 2010). No clear consensus exists on the reasons for these declines. We expect that the assembly reported here will facilitate conservation genomic approaches, shedding light on this species' current status ( Todisco *et al.,* 2020). In particular, it will be a valuable resource for any future reintroductions, monitoring, and other local conservation efforts.

## Genome sequence report

**Figure 1.**
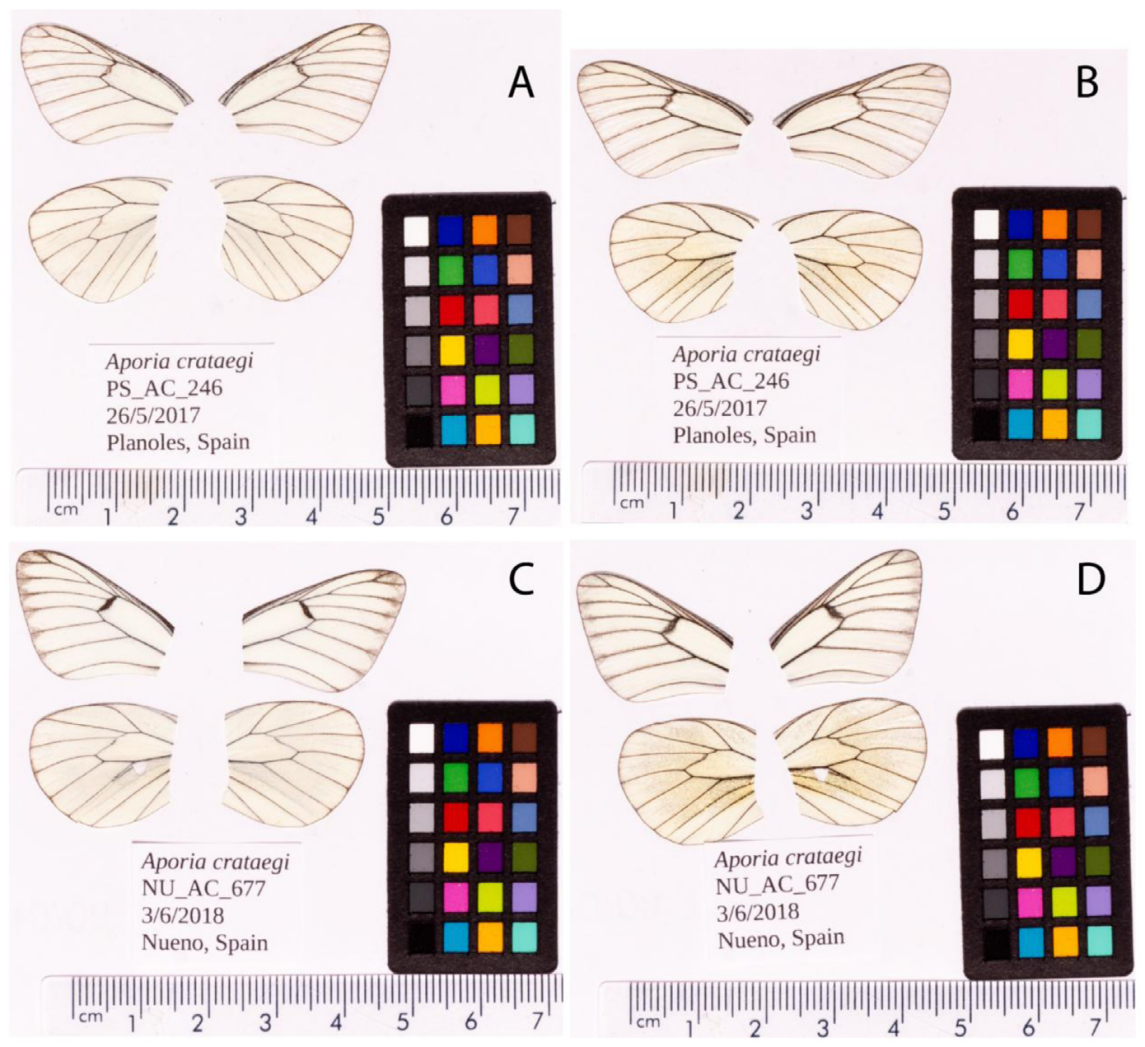
Fore and hind wings of the *Aporia crataegi* specimens used for sequencing. Dorsal ( **A**) and ventral ( **B**) surface view of wings from specimen PS_AC_246 (ilApoCrat1) from Planoles, Spain, used to generate Pacific Biosciences and 10X genomics data. Dorsal ( **C**) and ventral ( **D**) surface view of wings from specimen NU_AC_677 (ilApoCrat2) from Nueno, Spain, used to generate RNA-Seq data.

The genome was sequenced from a single female *A. crataegi* ( Figure 1) collected from Planoles Station, Catalunya, Spain (latitude 42.3136, longitude 2.0996). A total of 101-fold coverage in Pacific Biosciences single-molecule circular consensus (HiFi) long reads and 147-fold coverage in 10X Genomics read clouds were generated. Primary assembly contigs were scaffolded with chromosome conformation Hi-C data. Manual assembly curation corrected 4 missing/misjoins and removed 5 haplotypic duplications, reducing the assembly length by 0.37% and the scaffold number by 7.14%.

**Figure 2.**
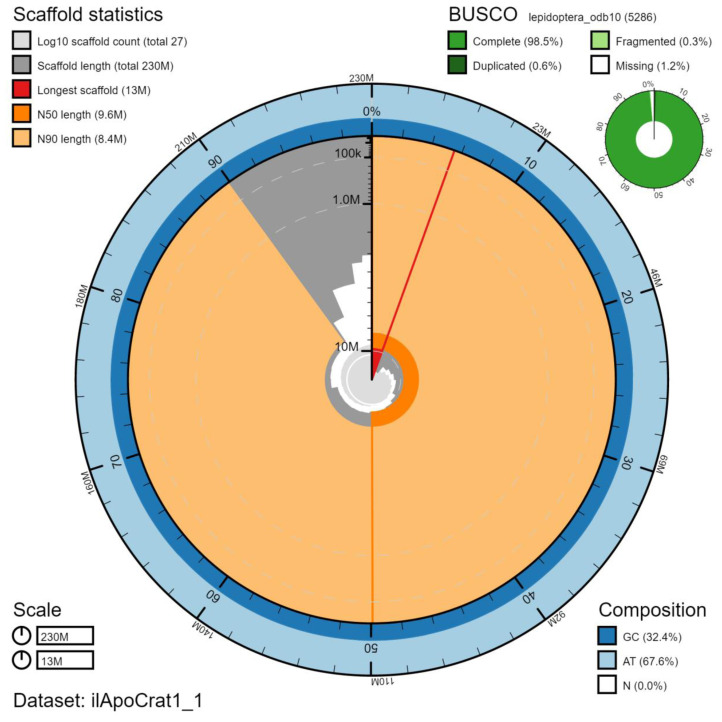
Genome assembly of *Aporia crataegi*, ilApoCrat1.1: metrics. The BlobToolKit Snailplot shows N50 metrics and BUSCO gene completeness. The main plot is divided into 1,000 size-ordered bins around the circumference with each bin representing 0.1% of the 229,694,036 bp assembly. The distribution of chromosome lengths is shown in dark grey with the plot radius scaled to the longest chromosome present in the assembly (12,847,094 bp, shown in red). Orange and pale-orange arcs show the N50 and N90 chromosome lengths (9,626,953 and 8,364,946 bp), respectively. The pale grey spiral shows the cumulative chromosome count on a log scale with white scale lines showing successive orders of magnitude. The blue and pale-blue area around the outside of the plot shows the distribution of GC, AT and N percentages in the same bins as the inner plot. A summary of complete, fragmented, duplicated and missing BUSCO genes in the lepidoptera_odb10 set is shown in the top right. An interactive version of this figure is available at https://blobtoolkit.genomehubs.org/view/ilApoCrat1.1/dataset/ilApoCrat1_1/snail.

The final assembly has a total length of 230 Mb in 26 sequence scaffolds with a scaffold N50 of 25.5 Mb ( Table 1). The complete assembly sequence was assigned to 26 chromosomal-level scaffolds, representing 25 autosomes (numbered by sequence length), and the Z sex chromosome ( Figure 2– Figure 5; Table 2). The assembly has a BUSCO v5.1.2 ( Manni *et al.,* 2021) completeness of 98.5% (single 97.8%, duplicated 0.6%) using the lepidoptera_odb10 reference set (n=5286). While not fully phased, the assembly deposited is of one haplotype. Contigs corresponding to the second haplotype have also been deposited.

**Table 1.** Genome data for *Aporia crataegi*, ilApoCrat1.1.

*Project accession data*	
Assembly identifier	ilApoCrat1.1
Species	*Aporia crataegi*
Specimen	ilApoCrat1 (genome assembly); ilApoCrat2 (RNA-Seq)
NCBI taxonomy ID	NCBI:txid129397
BioProject	PRJEB45674
BioSample ID	SAMEA7523355
Isolate information	Male, whole organism (ilApoCrat1); male, thorax (ilApoCrat2)
*Raw data accessions*	
PacificBiosciences SEQUEL II	ERR6544652
10X Genomics Illumina	ERR6363316-ERR6363319
Hi-C Illumina	ERR6363321
Illumina polyA RNA-Seq	ERR6363320
*Genome assembly*	
Assembly accession	GCA_912999735.1
*Accession of alternate haplotype*	GCA_912999795.1
Span (Mb)	230
Number of contigs	28
Contig N50 length (Mb)	9.6
Number of scaffolds	26
Scaffold N50 length (Mb)	9.6
Longest scaffold (Mb)	12.8
BUSCO * genome score	C:98.5%[S:97.8%,D:0.6%],F:0.3%,M:1.2%,n:5286
*Genome annotation*	
Number of protein-coding genes	10,860
Average length of coding sequence (bp)	1597.20
Average number of exons per transcript	8.23
Average exon size (bp)	259.64
Average intron size (bp)	1337.70

*BUSCO scores based on the lepidoptera_odb10 BUSCO set using v5.1.2. C= complete [S= single copy, D=duplicated], F=fragmented, M=missing, n=number of orthologues in comparison. A full set of BUSCO scores is available at https://blobtoolkit.genomehubs.org/view/ilApoCrat1.1/dataset/ilApoCrat1_1/busco.

**Figure 3.**
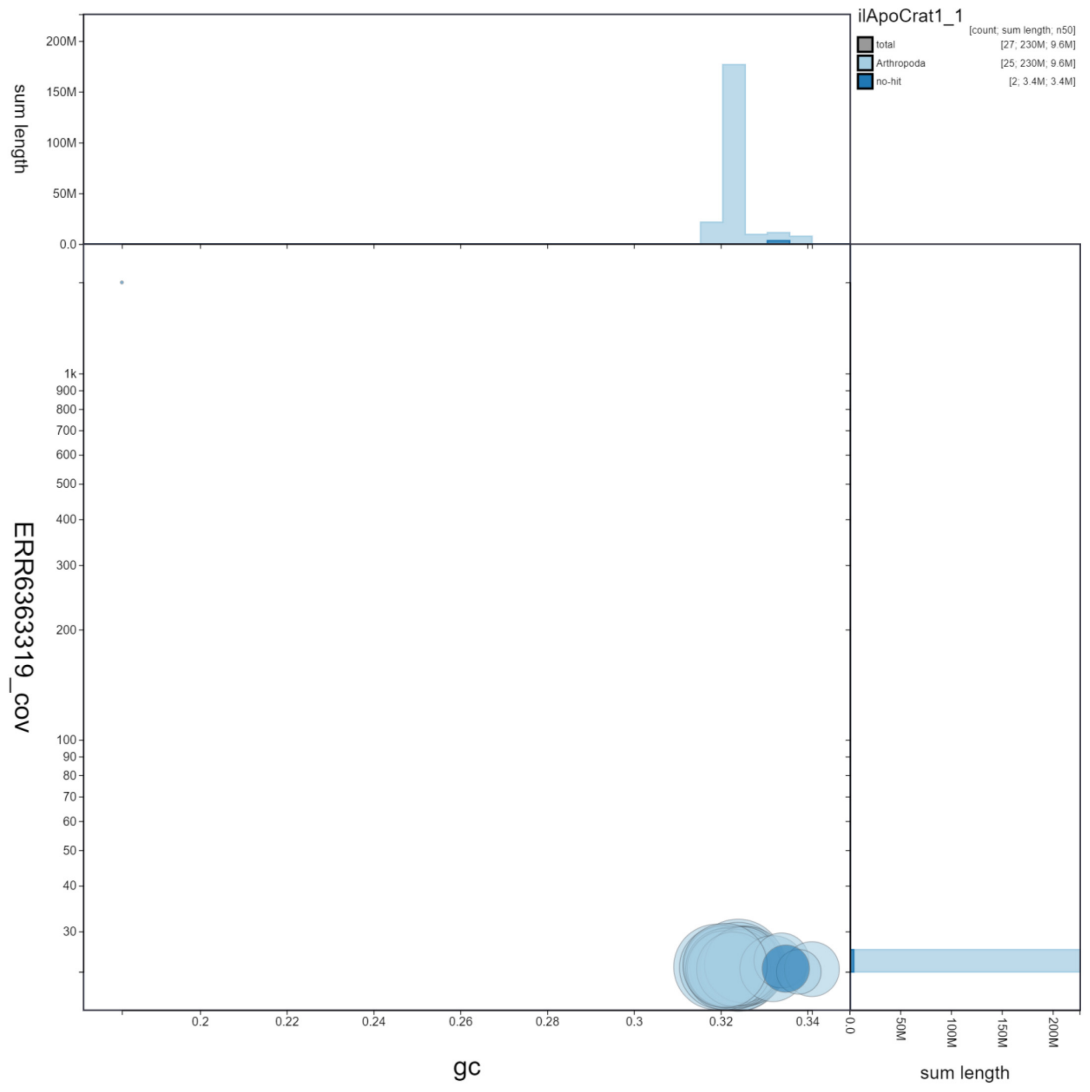
Genome assembly of *Aporia crataegi*, ilApoCrat1.1: GC coverage. BlobToolKit GC-coverage plot. Scaffolds are coloured by phylum. Circles are sized in proportion to scaffold length. Histograms show the distribution of scaffold length sum along each axis. An interactive version of this figure is available at https://blobtoolkit.genomehubs.org/view/ilApoCrat1.1/dataset/ilApoCrat1_1/blob.

**Figure 4.**
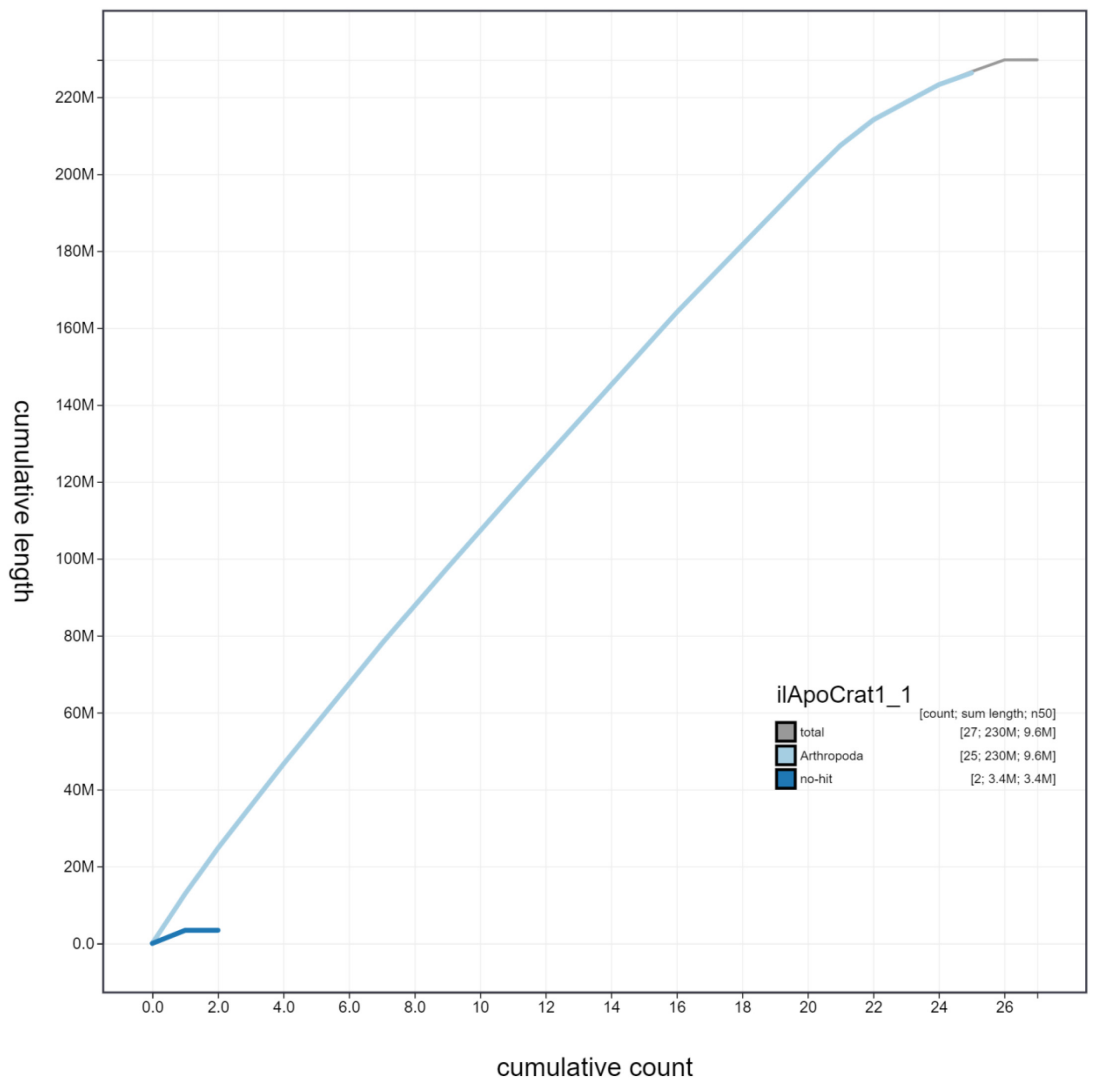
Genome assembly of *Aporia crataegi*, ilApoCrat1.1: cumulative sequence. BlobToolKit cumulative sequence plot. The grey line shows cumulative length for all scaffolds. Coloured lines show cumulative lengths of scaffolds assigned to each phylum using the buscogenes taxrule. An interactive version of this figure is available at https://blobtoolkit.genomehubs.org/view/ilApoCrat1.1/dataset/ilApoCrat1_1/cumulative.

**Figure 5.**
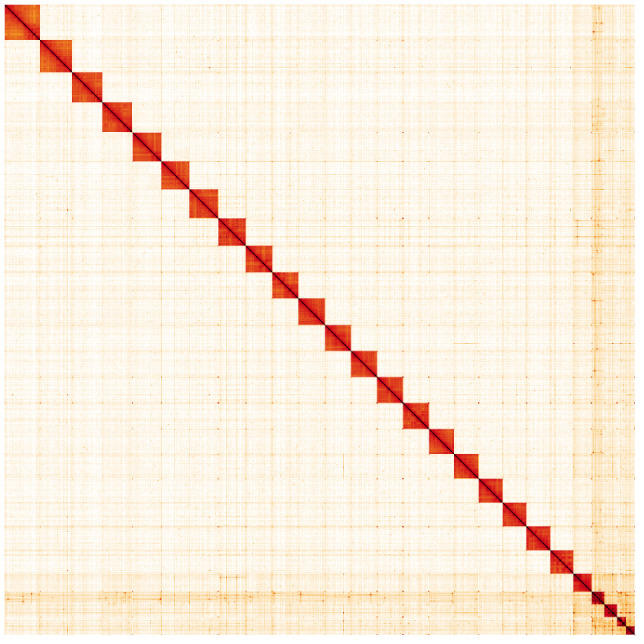
Genome assembly of *Aporia crataegi*, ilApoCrat1.1: Hi-C contact map. Hi-C contact map of the ilApoCrat1.1 assembly, visualised in HiGlass. Chromosomes are shown in size order from left to right and top to bottom.

**Table 2.** Chromosomal pseudomolecules in the genome assembly of *Aporia crataegi*, ilApoCrat1.1.

INSDC accession	Chromosome	Size (Mb)	GC%
OU538729.1	1	12.85	32.4
OU538730.1	2	11.89	32.4
OU538731.1	3	11.06	31.9
OU538733.1	4	10.56	32.3
OU538734.1	5	10.37	32.0
OU538735.1	6	10.35	32.1
OU538736.1	7	9.98	32.5
OU538737.1	8	9.76	32.4
OU538738.1	9	9.64	32.2
OU538739.1	10	9.63	32.5
OU538740.1	11	9.51	32.1
OU538741.1	12	9.47	32.2
OU538742.1	13	9.42	32.1
OU538743.1	14	9.39	32.6
OU538744.1	15	9.37	32.4
OU538745.1	16	8.83	32.5
OU538746.1	17	8.79	32.4
OU538747.1	18	8.74	32.1
OU538748.1	19	8.73	32.5
OU538749.1	20	8.36	32.3
OU538750.1	21	6.60	33.2
OU538751.1	22	4.59	33.4
OU538752.1	23	4.58	34.1
OU538753.1	24	3.40	33.5
OU538754.1	25	3.03	33.8
OU538732.1	Z	10.79	32.1
OU538755.1	MT	0.02	18.7

## Genome annotation report

The ilApoCrat1.1 genome has been annotated using the Ensembl rapid annotation pipeline ( Table 1; https://rapid.ensembl.org/Aporia_crataegi_GCA_912999735.1/). The resulting annotation includes 17,867 transcribed mRNAs from 10,860 protein-coding and 1,089 non-coding genes. There are 1.54 coding transcripts per gene and 8.23 exons per transcript.

## Methods

### Sample acquisition and nucleic acid extraction

A male *A. crataegi* specimen (ilApoCrat1, genome assembly) was collected from Planoles Station, Catalunya, Spain (latitude 42.3136, longitude 2.0996) using a net by Konrad Lohse, who also identified the specimen, and Alex Hayward. A second male *A. crataegi* specimen (ilApoCrat2, RNA-Seq) was collected from Nueno, Aragon, Spain (latitude 42.27, longitude -0.45) using a net by Sam Ebdon and Alexander Mackintosh. This specimen was also identified by Konrad Lohse. The samples were snap-frozen at -80°C. Permissions for field sampling were obtained from the Gobierno de Aragon (INAGA/500201/24/2018/0614 to Karl Wotton) and the Generalitat de Catalunya (SF/639).

DNA was extracted from the whole organism of ilApoCrat1 at the Wellcome Sanger Institute (WSI) Scientific Operations core from the whole organism using the Qiagen MagAttract HMW DNA kit, according to the manufacturer’s instructions. RNA (from the thorax of ilApoCrat2) was extracted in the Tree of Life Laboratory at the WSI using TRIzol, according to the manufacturer’s instructions. RNA was then eluted in 50 μl RNAse-free water and its concentration RNA assessed using a Nanodrop spectrophotometer and Qubit Fluorometer using the Qubit RNA Broad-Range (BR) Assay kit. Analysis of the integrity of the RNA was done using Agilent RNA 6000 Pico Kit and Eukaryotic Total RNA assay.

### Sequencing

Pacific Biosciences HiFi circular consensus and 10X Genomics read cloud DNA sequencing libraries were constructed according to the manufacturers’ instructions. Poly(A) RNA-Seq libraries were constructed using the NEB Ultra II RNA Library Prep kit. DNA and RNA sequencing was performed by the Scientific Operations core at the WSI on Pacific Biosciences SEQUEL II (HiFi), Illumina HiSeq X (10X) and Illumina HiSeq 4000 (RNA-Seq) instruments. Hi-C data were also generated from remaining whole organism tissue of ilApoCrat1 using the Arima v2 Hi-C kit and sequenced on an Illumina NovaSeq 6000 instrument.

### Genome assembly

Assembly was carried out with Hifiasm ( Cheng *et al.*, 2021); haplotypic duplication was identified and removed with purge_dups ( Guan *et al.,* 2020). One round of polishing was performed by aligning 10X Genomics read data to the assembly with longranger align, calling variants with freebayes ( Garrison & Marth, 2012). The assembly was then scaffolded with Hi-C data ( Rao *et al.*, 2014) using SALSA2 ( Ghurye *et al.*, 2019). The assembly was checked for contamination as described previously ( Howe *et al.*, 2021). Manual curation ( Howe *et al.*, 2021) was performed using HiGlass ( Kerpedjiev *et al.,* 2018) and Pretext. The mitochondrial genome was assembled using MitoHiFi ( Uliano-Silva *et al.,* 2021), which performed annotation using MitoFinder ( Allio *et al.,* 2020). The genome was analysed and BUSCO scores generated within the BlobToolKit environment ( Challis *et al.,* 2020). Table 3 contains a list of all software tool versions used, where appropriate.

**Table 3.** Software tools used.

Software tool	Version	Source
Hifiasm	0.12-r304	Cheng *et al.,* 2021
purge_dups	1.2.3	Guan *et al.,* 2020
SALSA2	2.2	Ghurye *et al.,* 2019
longranger align	2.2.2	https://support.10xgenomics.com/ genome-exome/software/pipelines/ latest/advanced/other-pipelines
freebayes	1.3.1-17-gaa2ace8	Garrison & Marth, 2012
MitoHiFi	2	Uliano-Silva *et al.,* 2021
HiGlass	1.11.6	Kerpedjiev *et al.,* 2018
PretextView	0.2.x	https://github.com/wtsi-hpag/ PretextView
BlobToolKit	2.6.4	Challis *et al.,* 2020

## Data availability

European Nucleotide Archive: Aporia crataegi (black veined white). Accession number PRJEB45674; https://identifiers.org/ena.embl/PRJEB45674.

The genome sequence is released openly for reuse. The *A. crataegi* genome sequencing initiative is part of the Darwin Tree of Life (DToL) project. All raw sequence data and the assembly have been deposited in INSDC databases. Raw data and assembly accession identifiers are reported in Table 1.

## Author information

Members of the Darwin Tree of Life Barcoding collective are listed here: https://doi.org/10.5281/zenodo.5744972.

Members of the Wellcome Sanger Institute Tree of Life programme are listed here: https://doi.org/10.5281/zenodo.5744840.

Members of Wellcome Sanger Institute Scientific Operations: DNA Pipelines collective are listed here: https://doi.org/10.5281/zenodo.5746904.

Members of the Tree of Life Core Informatics collective are listed here: https://doi.org/10.5281/zenodo.5743293.

Members of the Darwin Tree of Life Consortium are listed here: https://doi.org/10.5281/zenodo.5638618.
