# Supplementary material for: The genome sequence of the Black-veined White butterfly, Aporia crataegi (Linnaeus, 1758)
Source: Wellcome Open Res. 2025 Feb 21;7:81. Originally published 2022 Mar 8. [Version 2] doi: 10.12688/wellcomeopenres.17709.2 (PMC11872023; doi:10.12688/wellcomeopenres.17709.2)
Supplement: Peer review history

# Reviewer report: Reviewer response for version 2

**DOI:** 10.21956/wellcomeopenres.23182.r119364

**Published:** 2025-03-01

**Authors:** Ryan Laurent

**Affiliations:** 1 University of Colorado, Boulder, USA

This is a concise and clear report on the genomic sequencing of Aporia crataegi following in the tradition of Wellcome Open Research genome data notes. I do not have anything to add but the correct use of plural after "RNA data" followed by were (not "was") is a necessary edit.

Are sufficient details of methods and materials provided to allow replication by others?

Yes

Is the rationale for creating the dataset(s) clearly described?

Yes

Are the datasets clearly presented in a useable and accessible format?

Yes

Are the protocols appropriate and is the work technically sound?

Yes

Reviewer Expertise:

phylogenetics of Lepidoptera

I confirm that I have read this submission and believe that I have an appropriate level of expertise to confirm that it is of an acceptable scientific standard.

# Reviewer report: Reviewer response for version 1

**DOI:** 10.21956/wellcomeopenres.19596.r52406

**Published:** 2022-10-17

**Authors:** Olli-Pekka Smolander

**Affiliations:** 1 Department of Chemistry and Biotechnology, Tallinn University of Technology, Tallinn, Estonia

The authors present the documentation of a genome sequencing and assembly of a single individual of *A. crataegi. *In general the work and results are reported with good rigour. However, there are a few points which in my opinion should be improved.

- In the abstract it is said that the assembly is from an individual male *A.crataegi. *However, in the Genome sequence report, it is said that the genome was sequenced from a single female. As it clearly cannot be both, please correct one or the other. To me it seems that the male would be the correct one.
- It might be beneficial to report also the length distributions of the transcripts.
- As manufacturers instructions may change, or there may be several options to choose from, it should be reported with more detail how the sequencing libraries were made. In the current for, it is not possibly to replicate work with certainty.
- For the RNA-Seq and Hi-C, it would be interesting to see the amounts of the generated data. Similarly, for the PacBio and 10x read clouds, the amount of data would be more exact way to report than the average coverage.
- Genome assembly methodology should be reported in somewhat increased detail. If there are modifications to default parameters for used software, those should be reported, or if default parameters are used, it should also be mentioned. Similarly, it would be good to report how many contigs or scaffolds were produced in each stage. From the description it is not entirely clear how the polishing was made after calling the variants, i.e., how was the consensus sequence produced?

Are sufficient details of methods and materials provided to allow replication by others?

No

Is the rationale for creating the dataset(s) clearly described?

Yes

Are the datasets clearly presented in a useable and accessible format?

Yes

Are the protocols appropriate and is the work technically sound?

Yes

Reviewer Expertise:

Genomics research

I confirm that I have read this submission and believe that I have an appropriate level of expertise to state that I do not consider it to be of an acceptable scientific standard, for reasons outlined above.

# Reviewer report: Reviewer response for version 1

**DOI:** 10.21956/wellcomeopenres.19596.r52410

**Published:** 2022-10-17

**Authors:** Linlin Zhang

**Affiliations:** 1 CAS and Shandong Province Key Laboratory of Experimental Marine Biology & Center of Deep Sea Research, Center for Ocean Mega-Science, Institute of Oceanology, Chinese Academy of Sciences, Qingdao, China

This article mainly reports a genome resource of the butterfly *Aporia crataegi*. At first, it gives a brief introduction of *Aporia crataegi*’s habitats, distribution and an ecological issue that this butterfly faced. The butterfly disappeared from the British Isles around 1925 and failed to be reintroduced. The abundance of *Aporia crataegi* is declining in many countries. Then, it describes the genome assembly results in detail including materials, sequencing methods and several basic data of the genome. Overall, it provides a high quality genome of *Aporia crataegi* with 26 chromosomal-level scaffolds and a high Busco score, however, if researchers want to give some ideas from a genomic perspective on this butterfly’s current status, they still need to do more research about the *Aporia* *crataegi’*s genome.

 There are two mistakes in this article needed to be corrected:

1. In the “Genome sequence report” part, the first sentence “a single female” should be “a single male”.
2. In table 1, “Scaffold N50 length (Mb)” should be 25.5 rather than 9.6.

There are two suggestions:

1. You can provide a Busco score of the gene set to tell us the quality of the genome annotation result.
2. Do all the softwares use default parameters? If not, specific parameter settings should be given.

Are sufficient details of methods and materials provided to allow replication by others?

Yes

Is the rationale for creating the dataset(s) clearly described?

Yes

Are the datasets clearly presented in a useable and accessible format?

Yes

Are the protocols appropriate and is the work technically sound?

Yes

Reviewer Expertise:

EvoDevoGenomics

I confirm that I have read this submission and believe that I have an appropriate level of expertise to confirm that it is of an acceptable scientific standard.
